# ﻿A new *Bolitoglossa* (Amphibia, Caudata, Plethodontidae) from the Cordillera Oriental of Colombia

**DOI:** 10.3897/zookeys.1158.99077

**Published:** 2023-04-19

**Authors:** Yeny Rocio López-Perilla, Juan David Fernández-Roldán, Fabio Leonardo Meza-Joya, Guido Fabian Medina-Rangel

**Affiliations:** 1 Fundación Natura, Bogotá D.C., Colombia; 2 Grupo de Morfología y Ecología Evolutiva, Instituto de Ciencias Naturales, Universidad Nacional de Colombia, Bogotá D.C., Colombia; 3 Laboratorio de Anfibios, Instituto de Ciencias Naturales, Universidad Nacional de Colombia, Bogotá D.C., Colombia; 4 Grupo de Investigación en Biotecnología Industrial y Biología Molecular, Escuela de Biología, Universidad Industrial de Santander, Piedecuesta, Santander, Colombia; 5 Wildlife & Ecology, School of Natural Sciences, Massey University, Private Bag 11-222, Palmerston North, New Zealand; 6 Grupo Biodiversidad y Conservación, Instituto de Ciencias Naturales, Universidad Nacional de Colombia, Bogotá D.C., Colombia

**Keywords:** Biodiversity, coloration, phylogenetic systematics, salamanders, taxonomy, Biodiversidad, coloración, salamandras, sistemática filogenética, taxonomía

## Abstract

A new salamander species of the genus *Bolitoglossa* is here described from the cloud forests of the western slopes of the Cordillera Oriental of Colombia, in the Cundinamarca department. The most salient characters of this new species are its numerous maxillary and vomerine teeth, its moderate webbing on hands and feet, its short and robust tail, and its chromatic variation. Based on molecular analyses this new species is assigned to the *adspersa* species group and its status established as the sister species of *B.adspersa*, with which it was previously confused. Lastly, the distribution, natural history, and conservation status of the new species are discussed.

## Abstract (es)

Describimos una nueva especie de salamandra del género *Bolitoglossa* proveniente de los bosques nublados de la vertiente occidental de la Cordillera Oriental de Colombia, en el departamento de Cundinamarca. Los caracteres más sobresalientes de esta nueva especie son sus numerosos dientes maxilares y vomerinos, su palmeadura moderada en pies y manos, su cola corta pero robusta, y su variación cromática. Basados en análisis moleculares asignamos esta nueva especie al grupo de especies *adspersa* y establecemos su estatus como especie hermana de *B.adspersa*, con la cual era previamente confundida. Finalmente, discutimos algunos aspectos de su distribución, historia natural y su estado de conservación.

## ﻿Introduction

*Bolitoglossa* Duméril, Bibron & Duméril, 1854 is currently the largest and most diverse genus of salamanders (Amphibia: Caudata) with a total of 138 species recorded from northeastern Mexico to central Bolivia (Frost 2023). However, the actual diversity of *Bolitoglossa* in South America may be underestimated given the existence of cryptic forms (Jaramillo et al. 2020). Colombia currently has 24 species (Frost 2023), of which only seven have been described in this century, while the remainder are descriptions prior to 1973 (Frost 2023). As for the phylogenetic relationships for the species of the country, little is known; there are some works that include species distributed in the Colombian Amazon (Cusi et al. 2020; Jaramillo et al. 2020), as well as phylogenies presented in the most recent descriptions of species in the country (Acevedo et al. 2013; Meza-Joya et al. 2017).

The genus *Bolitoglossa* has its highest diversity in the Cordillera Oriental (Meza-Joya et al. 2017), where a total of 11 species have been described: *B.adspersa* (Peters, 1863), *B.altamazonica* (Cope, 1874), *B.capitana* Brame & Wake, 1963, *B.guaneae* Acosta-Galvis & Gutierrez, 2012, *B.leandrae* Acevedo, Wake, Márquez, Silva, Franco & Amézquita, 2013, *B.lozanoi* Acosta-Galvis & Restrepo, 2001, *B.nicefori* Brame & Wake, 1963, *B.palmata* (Werner, 1897), *B.pandi* Brame & Wake, 1963, *B.tamaense* Acevedo, Wake, Márquez, Silva, Franco & Amézquita, 2013, and *B.yariguiensis* Meza-Joya, Hernández-Jaimes & Ramos-Pallares, 2017.

These ‘mushroom-tongued’ or ‘tropical lungless’ salamanders, as they are commonly known, are characterized by lacking a sublingual fold; having a very long and rapidly projected tongue; a tendency towards tarsal reductions; extensive webbing associated with climbing behavior; and fully terrestrial to arboreal habits (Wake and Lynch 1976; Köhler 2011; Angarita-Sierra et al. 2020; Ponssa et al. 2022).These salamanders inhabit a variety of ecosystems, from lowland rainforests to highland areas, where they are particularly diverse in montane cloud forests and less so in paramo ecosystems (Köhler 2011).

In this paper, we describe a new species of *Bolitoglossa* using morphological and molecular data, associated with the existing remnants of Andean montane forests on the western flank of the Cordillera Oriental, and we compared it with other known species of the genus in Cundinamarca, Colombia.

## ﻿Materials and methods

### ﻿Specimen collection and fieldwork

**Figure 1.**
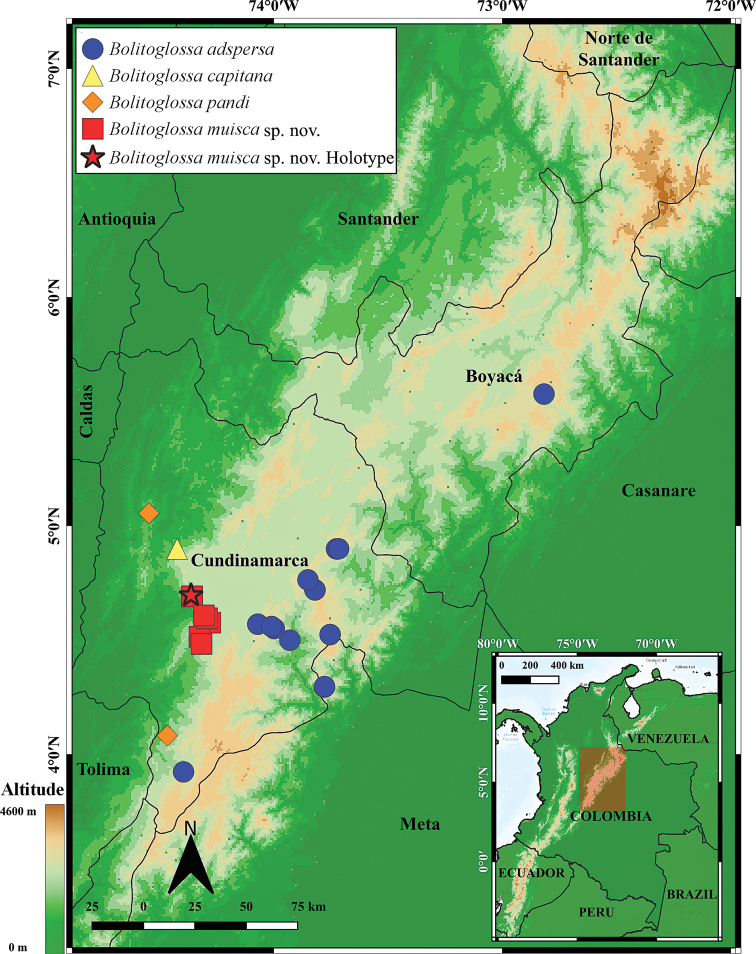
Map of Colombia showing the distribution records of *Bolitoglossamuisca* and other sympatric *Bolitoglossa* species in the Cordillera Oriental.

The holotype and most of the paratypes were collected within vereda Roble Hueco, Bojacá municipality, Cundinamarca department, Colombia (4.6963, -74.3624, 2630 m a.s.l.; Fig. 1). Specimens were captured by hand using free searches over two 10-day field trips, kept in plastic bags until weighed and photographed, and then euthanized by applying 2% lidocaine gel. Tissue samples were obtained from the tail or liver of individuals and preserved in absolute ethanol. Specimens were then fixed in 10% formaldehyde and then stored in 70% ethanol. All specimens were deposited at Colección de Anfibios, Instituto de Recursos Biológicos Alexander von Humboldt, Villa de Leyva, Boyacá, Colombia (**IAvH**).

### ﻿Morphology and taxonomy

Measurements and counts of morphological characters were taken using a Neiko digital caliper rounded to the nearest 0.1 mm under a Leica Stemi 2000 stereoscope, using the diagrams in Bingham et al. (2018) as a model. The following morphological traits were analyzed for all specimens: snout-vent length (**SVL**); head length (**HL**); head width (**HW**); tail length (**TL**); maxillary teeth (**MT**); vomerine teeth (**VT**); and additional measurements only for the description of the holotype: interorbital distance (**IOD**); eye diameter (**EYD**); snout length (**SNL**). Color descriptions are based on field notes and photographs of preserved specimens using the color catalogue of Köhler (2012). Format of diagnosis and description follows Meza-Joya et al. (2017). Species comparisons were made following Brame and Wake (1963) or their original descriptions for those species described after 1963, as well as by examining *Bolitoglossa* specimens housed at Colección de Anfibios, Instituto de Recursos Biológicos Alexander von Humboldt, Villa de Leyva, Boyacá, Colombia (**IAvH**), Laboratorio de Anfibios, Instituto de Ciencias Naturales, Universidad Nacional de Colombia, Bogotá D.C., Colombia (**ICN**), Colección de Anfibios, Museo La Salle, Universidad de La Salle, Bogotá D.C., Colombia (**MLS**) and Colección Herpetológica of Universidad Industrial de Santander, Bucaramanga, Colombia (**UIS**).

### ﻿Molecular and phylogenetic analyses

We extracted whole genomic DNA from liver or muscle tissue of specimens of *Bolitoglossa* preliminary identified as *B.adspersa*, using the DNeasy Blood & Tissue Kit (Qiagen, #69506). Extracted DNA samples were amplified by PCR for the partial non-coding 16S rRNA (16S ≈ 517 bp) and the protein-coding cytochrome b (cyt b ≈ 742 bp) mitochondrial genes, using the primers 16Sar-L and 16Sbr-H (Palumbi et al. 1991) and MVZ15 and MVZ16 (Moritz et al. 1992), respectively. Amplification protocols (reaction mix and thermocycler programs) are as described in Meza-Joya et al. (2017). The amplicons were cleaned and then sequenced at Macrogen Inc. (Seoul, Korea) by capillary electrophoresis using an ABI3730 genetic analyzer. The partial sequences obtained were visualized, cleaned, and assembled with Geneious v. 9.1.6 (Kearse et al. 2012); we only used sequences with a quality score higher than 90%. DNA sequences were deposited in GenBank (Appendix 1).

Homologous sequences from other *Bolitoglossa* in the *adspersa* group were downloaded from GenBank and compared with our molecular data. Representatives from other species groups within *Eladinea* (*epimela*, *schizodactyla*, and *subpalmata*) were used as outgroups (Appendix I). We also included partial sequences of the nuclear protein-coding recombination activating gene 1 (RAG1 ≈ 792 bp), a relatively well-sampled gene fragment, for other species of *Bolitoglossa*. We performed multiple alignments in MAFFT v. 7.304 (Katoh and Standley 2013) using the G-INS-i algorithm. Phylogenetic analyses were performed on the concatenated dataset. We inferred the best-fit partition scheme and the best-fit evolution models with PartitionFinder v. 2.1.1 (Lanfear et al. 2017) under the Bayesian Information Criterion (BIC). For this, we performed an exhaustive search of all possible partitioning schemes on our dataset, placing the 16S gene in a separate partition whereas protein-coding genes were further partitioned by codon position. We performed a maximum likelihood (ML) phylogenetic analysis on IQ-TREE 2.0 (Nguyen et al. 2015), running 10,000 ultrafast bootstrap pseudoreplicates for internal node support (Hoang et al. 2018). We also conducted a Bayesian Inference (BI) analysis with the software MrBayes 3.2.6 (Ronquist et al. 2012), using four chains on two runs for 10 million generations and a sampling frequency of 10,000 generations with a burn-in of 0.10. Stationarity was determined with the software Tracer 1.6 (Rambaut et al. 2018).

For species delimitation, we first calculated uncorrected pairwise genetic p-distances for the 16S and cyt b genes between the two distinct evolutionary lineages identified within *B.adspersa*, with 1,000 bootstrap replicates using MEGA X (Kumar et al. 2018). Then, we split genetic lineages into candidate species using the Automatic Barcode Gap Discovery (ABGD) method (Puillandre et al. 2012). This analysis was based on the 16S gene matrix, the best-represented gene in our dataset, using K2P distances, prior for maximum value of intraspecific divergence between 0.001 and 0.1, ten recursive steps, and a gap width of 1.5. We also used the tree-based method as implemented in the Species Delimitation plugin in Geneious (Masters et al. 2011), using the ML phylogeny as a guide tree to calculate the mean probability of the ratio of intra- to interspecific genetic distances for the initial two-species hypothesis within *B.adspersa* (Brame and Wake 1963).

## ﻿Results

### ﻿Molecular and phylogenetic analyses

**Figure 2.**
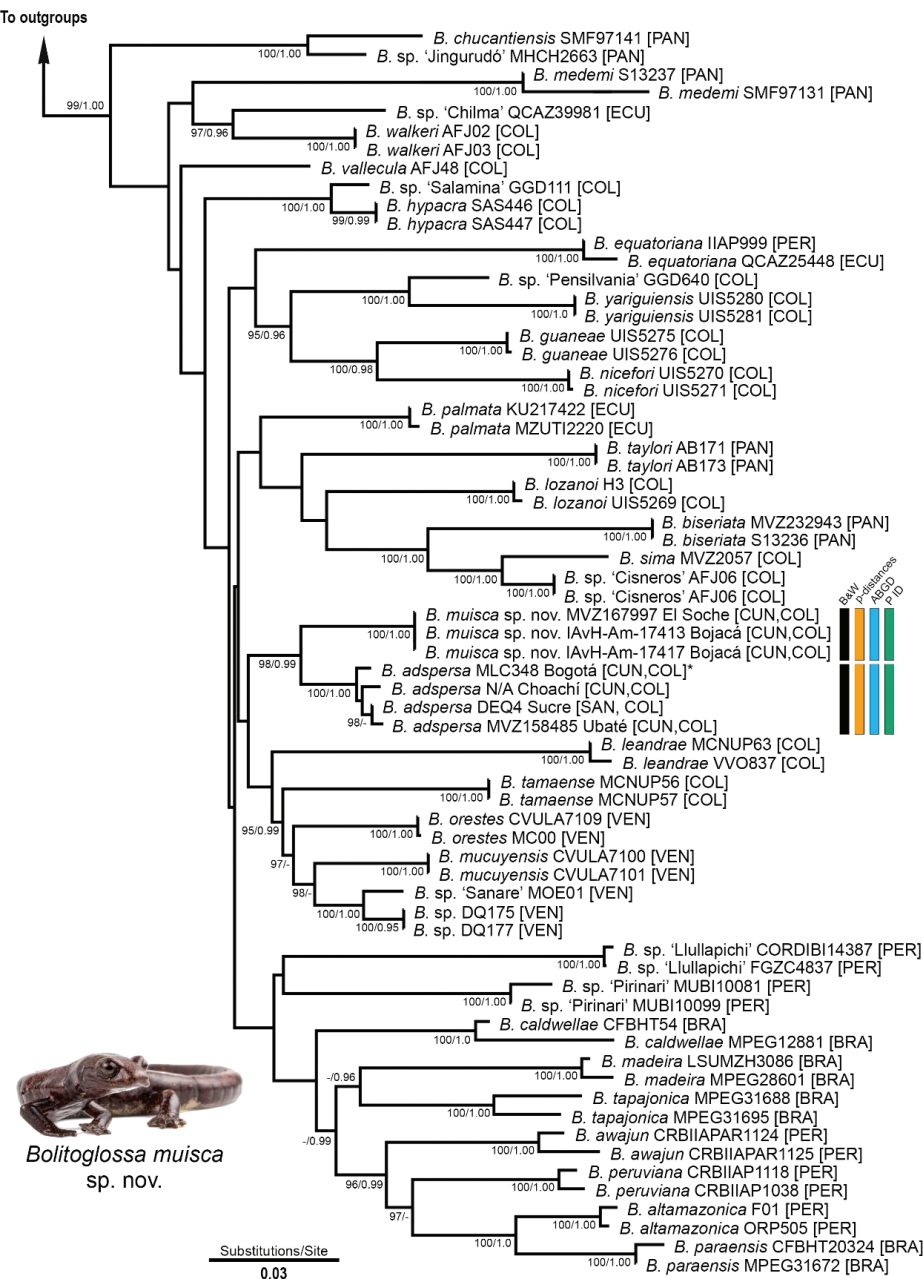
Maximum-likelihood tree of Bolitoglossa (Eladinea) adspersa group showing the phylogenetic position of *Bolitoglossamuisca*. Vertical bars indicate the two-species hypothesis within *Bolitoglossaadspersa* with the supported species partitions inferred using genetic data (p-distances, ABGD, and P ID). Support values for well-supported nodes correspond to ML ultrafast bootstrap (> 95) and Bayesian posterior probabilities (> 0.95), respectively. Photograph by JDF.

The concatenated data matrix contains 2,070 bp for 67 terminals (excluding outgroups) from 26 described species of the *Bolitoglossaadspersa* group, as well as nine candidate species and the new species described herein: 64 samples for 16S (516 bp), 45 for cyt b (759 bp), and 26 for RAG1 (795 bp). The best partition scheme (LnL = -14128.62, BIC = 29,677.40) for our concatenated dataset includes five subsets, each with an evolution model: (16S, cyt b pos1: GTR+I+G) (cyt b pos2: HKY+I+G) (cyt b pos3: GTR+G) (RAG1 pos1, RAG1 pos2: K81+I) (RAG1 pos3: HKY+G). The resulting topologies from the ML and BI were congruent; thus, here we present only the ML tree (Fig. 2).

The inferred phylogenetic relationships were largely consistent with those from recent studies (Meza-Joya et al. 2017; Cusi et al. 2020; Jaramillo et al. 2020), with incongruences likely resulting from differences in taxon sampling. The *Bolitoglossaadspersa* species group was rendered as monophyletic with significant support (UFB = 99, PP = 1.0). All species included in our analyses were monophyletic with strong support, yet their relationships remained largely unresolved. As expected, samples from the new species were within the *adspersa* group, forming a well-supported clade (UFB = 100, PP = 1.0) sister to samples of *B.adspersa* from its type-locality, Bogotá, Cundinamarca department, Colombia, and surroundings, with significant support (UFB = 98, PP = 0.99). This clade was recovered as a sister to a clade grouping species from northeastern Colombia and Venezuela, but this relationship was poorly supported. GenBank sequences of a specimen from El Soche, Cundinamarca, Colombia, identified as *Bolitoglossa* sp. 1 (MVZ 167947, corrected here as MVZ 167997 based on the actual number on the MVZ catalog) by Parra-Olea et al. 2004, correspond with the new species described here. This specimen has long been recognized as different from *adspersa* (Hanken and Wake 1982; Parra-Olea et al. 2004).

With respect to species delimitation, all methods supported the two-species hypothesis within *B.adspersa* (Brame & Wake, 1963). Uncorrected pairwise p-distances between these two sister lineages were 2.1% (± 0.003%) for 16S and 7.0% for cyt b. The ABGD analysis resulted in five partitions separating the data into five (P ≤ 0.0028) or two putative species (P ≥ 0.0046), yet the new species described here was recovered as a candidate taxon in all partitions. Taxonomic distinctiveness for the new species was also supported under either relaxed or strict tree-based criteria (P ID Liberal = 1.00, CI = 0.86–1.00; P ID Strict = 0.79, CI = 0.62–0.97) with significant support: Rosenberg’s P_AB_ statistics = 0.01 and P_RD_ (randomly distinct) > 0.05. Furthermore, as it is shown below, morphological comparisons consistently support the recognition of this lineage as a new species.

#### ﻿ Bolitoglossa muisca sp. nov.

Taxon classificationAnimaliaCaudataPlethodontidae

A20FF296-692C-52CB-A46D-56B87934B936

https://zoobank.org/63BA9285-C4FE-45B0-B828-BDB37F4E06BF

**Figure 3.**
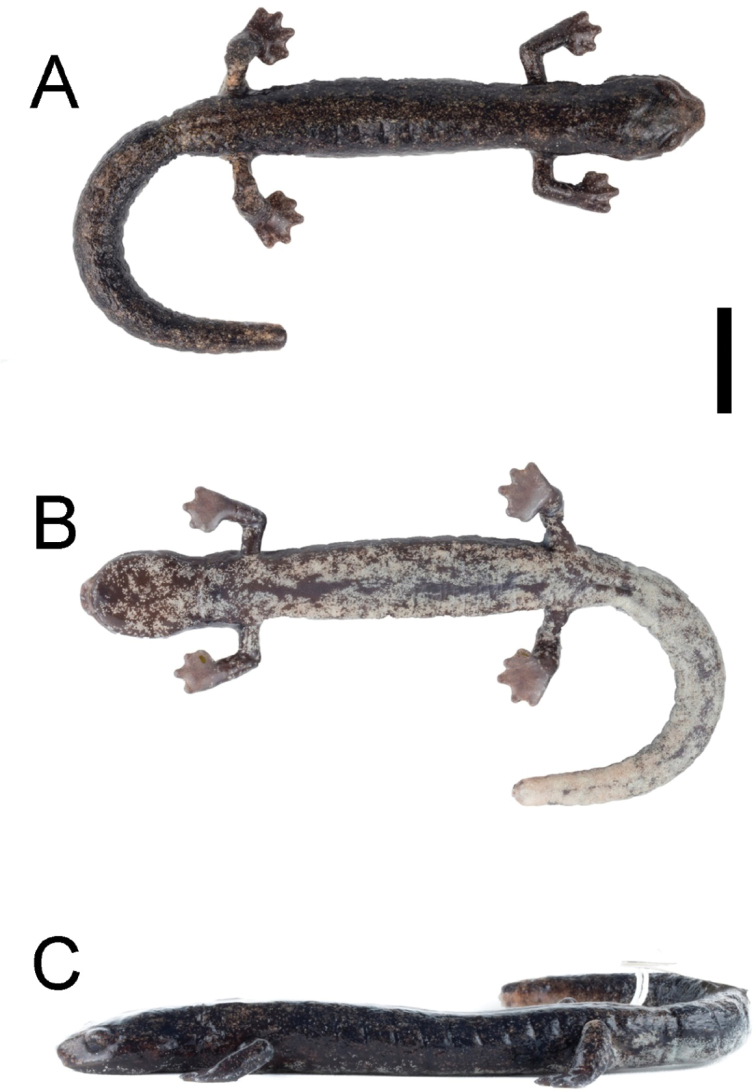
Holotype of *Bolitoglossamuisca* (IAvH-Am-17413) in **A** dorsal **B** ventral and **C** lateral views. Photographs by JDF. Scale bar: 10 mm.

**Figure 4.**
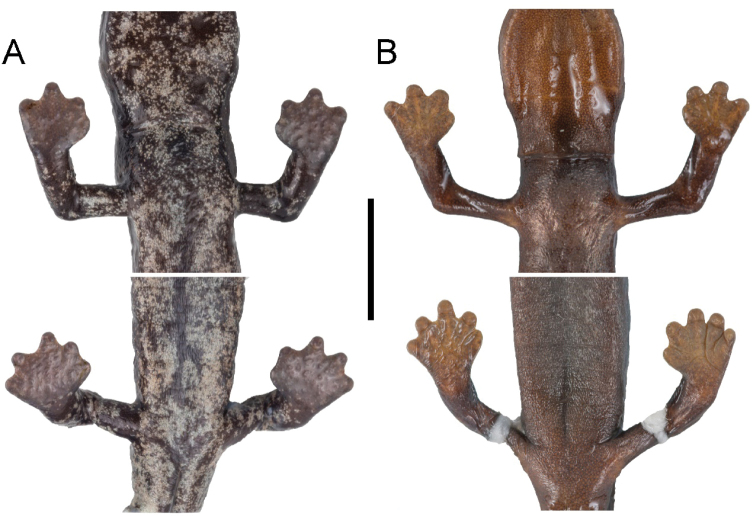
Ventral views of the hands and feet of **A***Bolitoglossamuisca* (IAvH-Am-17413) **B***Bolitoglossaadspersa* (ICN 4885). Photographs by JDF. Scale bar: 10 mm.

**Figure 5.**
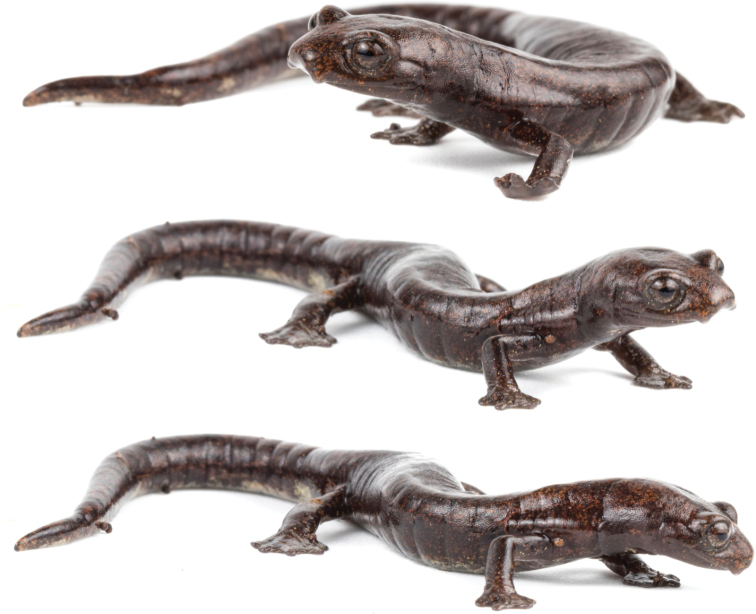
Paratype of *Bolitoglossamuisca* (IAvH-Am-17419) in life. Photographs by JDF.

**Figure 6.**
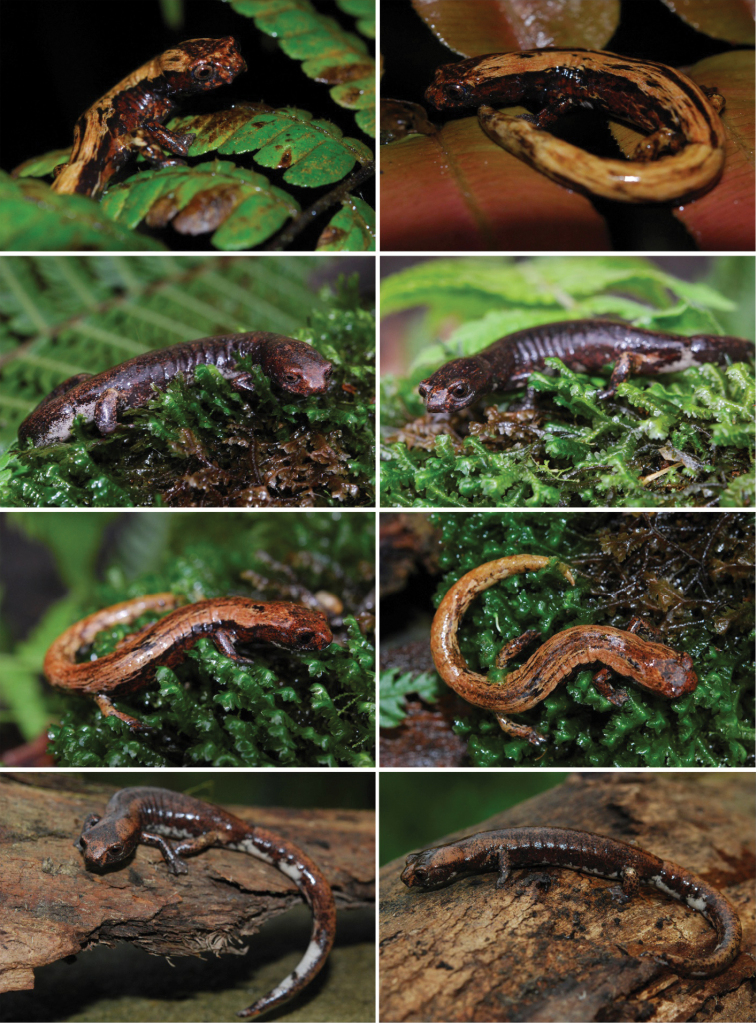
Chromatic variation of *Bolitoglossamuisca* in life. Notice some individuals bear white blotches on the ventral surfaces of the body and the tail. Photographs by YLP.

**Figure 7.**
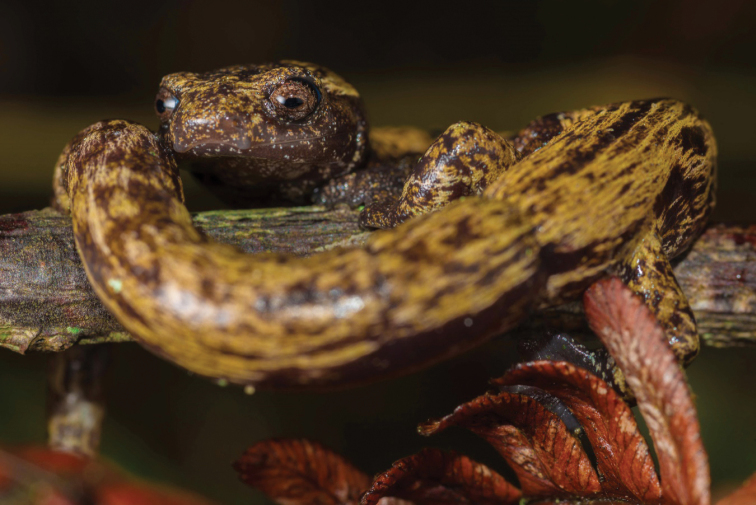
A non-captured individual of *Bolitoglossamuisca* from the surroundings of Reserva Chicaque, San Antonio del Tequendama, Cundinamarca, Colombia, photographed by JDF in October 2015. Notice the striking contrasting dark brown background coloration with ochre markings primarily on the dorsal surfaces of the body.

Figs 2 , 3 , 4 , 5 , 6 , 7 , Tables 1 , 2 Common English name: Muisca salamander Common Spanish name: Salamandra Muisca

##### Type material.

***Holotype*.**IAvH-Am-17413, an adult female from Finca La Esmeralda, vereda Roble Hueco, Bojacá municipality, Cundinamarca department, Colombia (4.6963, -74.3624, 2630 m a.s.l.), collected by Y.R. López-Perilla on 4 February 2021 (Fig. 1).

***Paratypes* (*n* = 8: 3 females, 5 males).**IAvH-Am-17417 (adult male), IAvH-Am-17419 (adult female), IAvH-Am-17421 (adult male) and IAvH-Am-17422 (adult male) from Finca La Esmeralda, vereda Roble Hueco, Bojacá municipality, Cundinamarca department, Colombia (4.6963, -74.3624, 2630 m a.s.l.), collected by Y.R. López-Perilla in February 2021 (Fig. 5). IAvH-Am-17423 (adult male), IAvH-Am-17425 (adult male) and IAvH-Am-17428 (adult female) from Finca Peñas Blancas, vereda Roble Hueco, Bojacá municipality, Cundinamarca department, Colombia (4.6916, -74.3581, 2390 m a.s.l.), collected by Y.R. López-Perilla in February 2021. IAvH-Am-17429 (adult female) from vereda Cascajal, Soacha municipality, Cundinamarca department, Colombia (4.5954, -74.2922, 2700 m a.s.l.), collected by G.F. Medina-Rangel in September 2022.

##### Referred specimens

**(*n* = 36: 7 females, 29 juveniles and subadults).**ICN 3544–48, five adult females from Hacienda ‘El Soche’, Granada municipality, Cundinamarca department, Colombia (4.5153, -74.3240, 2600 m a.s.l.) obtained by Rurithza Velandia in December 1977; specimen ICN 3545 is cleared and stained; MVZ 167997, a juvenile obtained by Pere Alberch on 17 December 1978. ICN 58245–58268, a batch of 23 juvenile and subadult specimens from Hacienda ‘La Tribuna’, vereda Noruega Alta, Silvania municipality, Cundinamarca department, Colombia (4.4836, -74.3203, 2700 m a.s.l.) obtained by Cesar Monguí in September 2008. ICN 60319–20, two juveniles from vereda Roquemonte, San Antonio del Tequendama municipality, Cundinamarca department, Colombia (4.6056, -74.3008, 2600 m a.s.l.) obtained by J.D. Fernández in August 2013. ICN 60321–22, a juvenile and an adult female (respectively) also from vereda Roquemonte, San Antonio del Tequendama municipality, Cundinamarca department, Colombia (4.6056, -74.3008, 2600 m a.s.l.) obtained by J.D. Fernández in March 2017. ICN 60323–25, two juveniles and an adult female (respectively) from vereda Roquemonte, San Antonio del Tequendama municipality, Cundinamarca department, Colombia (4.6056, -74.3008, 2600 m a.s.l.), also obtained by J.D. Fernández in January 2018.

##### Diagnosis.

*Bolitoglossamuisca* is a member the subgenus Eladinea and of the *adspersa* species group. The new species is characterized by the following morphological characters: a large-size body; a broad head; a rounded snout in dorsal and ventral views; a very thick postocular fold; a moderate subgular fold; smooth skin texture; moderately long limbs; moderate webbing on third finger and toe; and a short, robust tail.

Even though *Bolitoglossaadspersa* and *B.muisca* share their moderate webbing on hands and feet, we regard the former as having less webbing than the latter. Moreover, the tips of the fingers and toes are separated from the distal margin of the webbing, exposing their subcircular-shaped digits; unlike those of *B.muisca* (Fig. 4). The new species is slightly larger on average than *B.adspersa* (mean SVL 52.8±3.4 mm; range 33.0–72.1 mm; *n* = 22 vs. 45.0±3.4 mm; range 30.7–66.3 mm; *n* = 26), additionally, the tail of *B.adspersa* is thin and long in relation to the trunk but thick and short in relation to the trunk in *B.muisca* (Table 1). *Bolitoglossamuisca* differs from *B.capitana* by having moderately webbed hands and feet (vs. almost fully webbed hands and feet in *B.capitana*), by being overall smaller in size (mean SVL 52.8±3.4 mm; range 33.0–72.1 mm; *n* = 22 vs. 75.2±10.9 mm; range 59.2–85.5 mm; *n* = 5), by having fewer maxillary teeth (mean MT 24.4±3.0; range 14–36; *n* = 17 vs. 30.4±5.1; range 22–37; *n* = 5) by having fewer vomerine teeth (mean VT 35.1±4.0; range 24–53; *n* = 17 vs. 66.4±14.8; range 46–87; *n* = 5), and because the new species bears a faint, very small gular fold that is large, thick and notable in *B.capitana* (Cruz-Rodríguez et al. 2021: figs 13, 14). *Bolitoglossamuisca* differs from *B.pandi* because the tips of the third digit and toe of the latter are triangular and pointed in outline (vs. third digits and toes oval and webbed to a higher degree in *B.muisca*), by being a slightly larger species (mean SVL 52.8±3.4 mm; range 33.0–72.1 mm; *n* = 22 vs. 44.0±2.9 mm; range 35.9–52 mm; *n* = 12), and by having mostly smooth skin (vs. coarse skin in *B.pandi*); moreover, the tail of *B.pandi* tapers gradually and symmetrically from broad to slender antero-posteriorly, unlike that of the new species, which is slightly rectangular in outline, becoming abruptly wider than the base, and ending in a rounded tip (Table 1).

**Table 1.** Meristic data and morphological comparisons of *Bolitoglossaadspersa*, *B.capitana*, *B.muisca*, and *B.pandi*. For abbreviations see methods section.

Characters/Species	*B.adspersa*	*B.capitana*	*B.muisca*	*B.pandi*
VT	26.1±2.5 (18–38) [*n* = 17]	66.4±14.8 (46–87) [*n* = 5]	35.1±4 (24–53) [*n* = 17]	31.8±4 (21–42) [*n* = 12]
MT	17.6±1.5 (13–29) [*n* = 18]	30.4±5.1 (22–37) [*n* = 5]	24.4±3 (14–36) [*n* = 17]	18.3±2.2 (13–26) [*n* = 12]
SVL	45±3.4 (30.7–66.3) [*n* = 26]	75.2±10.9 (59.2–85.5) [*n* = 5]	52.8±3.4 (33–72.1) [*n* = 22]	44±2.9 (35.9–52) [*n* = 12]
TL	35±3.7 (21.2–54.2) [*n* = 26]	61.0±7.4 (53.2–70.8) [*n* = 4]	38.9±4.3 (11.6–55.5) [*n* = 22]	29.1±5.2 (15.4–42.8) [*n* = 11]
HW	6.6±0.4 (4.9–9.5) [*n* = 27]	10.9±1.5 (8.8–12.5) [*n* = 5]	7.9±0.4 (5.4–10.3) [*n* = 22]	6.6±0.5 (5.3–8.2) [*n* = 12]
HL	8.2±0.5 (5.8–10.2) [*n* = 27]	12.2 [*n* = 1]	9.1±0.7 (5.6–14.5) [*n* = 22]	7.47±0.6 (6.3–10.2) [*n* = 12]
TL/SVL	0.8±0.1 (0.6–1.1) [*n* = 11]	0.8±0.1 (0.7–1.1) [*n* = 4]	0.72±0.1 (0.2–0.9) [*n* = 22]	0.6±0.1 (0.34–1.1) [*n* = 11]
Webbing on third finger	Moderate	Extensive	Moderate	Moderate
Webbing on third toe	Moderate	Extensive	Moderate	Moderate
Postocular fold	Thick	Absent	Very thick	Absent
Subgular fold	Very thick	Thick	Moderate	Faint
Snout in dorsal and ventral view	Truncated	Truncated	Rounded	Truncated
Tail shape	Tapered	Tapered	Stout	Tapered

##### Description of the holotype.

An adult female (SVL = 61.3 mm) with a broad head (HW/SVL = 0.16); head longer than wide (HW/HL = 0.90); neck with a small, faint gular fold; snout short and truncated in profile, and dorsal view, but less so in ventral view (SNL = 3.1 mm); large eyes that do not extend beyond the outline of the head in dorsal view and smaller than interorbital distance (EYD = 3.18 mm, IOD = 3.37 mm); with a thick post ocular fold that extends past the posterior commissure of the eye onto the anterior margin of the gular fold; canthus rostralis subtle, small, rounded in outline; 33 maxillary teeth, 16 to the right and 17 to the left; vomerine teeth 25, these are not arranged in a single row but grouped towards the margins of the parasphenoid bone; with three premaxillary teeth that pierce the upper lip in males; nasolabial grooves well developed; moderate interdigital webbing on hands but third finger extends slightly further than the other fingers; toes with less interdigital webbing than fingers, toes II–V with less membrane than toe I; with subterminal pads on digits, digits in order of increasing length I<II<IV>III; toes I<II<III<IV>V; longest digits of hand and feet are subcircular (L3T and L3F), limbs relatively long (FL/SVL = 0.23, HLL/SVL = 0.23); tail not exceeding standard length (TL/SVL 0.93), narrower than the body at the base (posterior to the vent), slightly rectangular in outline, becoming abruptly wider than the base and ending in a rounded tip, but this condition is artefactual because the tip of the tail is missing; a long trunk (52.5 mm); with 13 costal grooves (Fig. 3). See Table 2 for meristic data of all type specimens.

**Table 2.** Meristic data and measurements of the type series of *Bolitoglossamuisca*.

Characters/Type series	IAvH-Am-17413 *	IAvH-Am-17417	IAvH-Am-17419	IAvH-Am-17421	IAvH-Am-17422	IAvH-Am-17423	IAvH-Am-17425	IAvH-Am-17428	IAvH-Am-17429
Sex	Female	Male	Female	Male	Male	Female	Male	Female	Female
SVL (mm)	61.3	50.6	60.3	58.1	57.4	72.1	54	51.5	65.9
TL (mm)	53.2	40.8	46.6	49.8	51.6	14.4^+^	45.8	36.5	42.6
HW (mm)	9.6	7.7	9.2	7.7	8.7	9.8	8.5	8.2	10.3
HL (mm)	10.6	8.9	10.7	9.4	9.7	11.6	9	9.1	14.5
HW/SVL	0.1	0.1	0.1	0.1	0.1	0.1	0.1	0.1	0.1
TL/SVL	0.8	0.8	0.7	0.8	0.9	--	0.8	0.7	0.6
VT	25	23	26	18	23	36	22	32	36
MT	33	24	28	36	44	44	36	41	44

* Denotes de holotype. ^+^ regenerated tail. -- No data. For abbreviations see Materials and methods section.

##### Coloration of the holotype in life.

The color pattern of the holotype is described based on a photograph taken the day after capture. The dorsal surfaces of the head, the body and the tail are Raw Umber (280), strongly speckled with Dark Salmon (59); white stipples on the lateral surface of the head; the flanks, dorsum, legs, and tail have an irregular thin white stripe; the iris is Light Sky Blue (191) with Pratt’s Rufous (72) reticulations. The throat and ventral surfaces are white with Raw Umber (280) speckles and reticulations; the ventral surfaces of the limbs and tails with some Light Orange Yellow (7) vermiculated; underside of hands and feet are Olive-Brown (278).

##### Coloration of the holotype in preservative.

The color pattern of the holotype was recorded after approximately five months stored in 70% ethanol. The dorsal surfaces of the head, the body and the tail are Raw Umber (280), strongly speckled with Dark Salmon (59); the flanks, dorsum, legs, and tail have an irregular Smoke Gray (266) stripe; the iris is Amber (51) with Orange Rufous (56) reticulations. The throat and ventral surface are Smoke Gray (266) with Raw Umber (280) speckles and reticulations; hands and feet soles are Grayish Horn (268) ventrally (Fig. 3).

##### Color variation.

The specimens IAvH-Am-17414–16 have the dorsal surfaces of the head, flanks, dorsum, front legs and vertebral band Orange-Rufous (56), strongly speckled with Raw Umber (280); paravertebral area, tail, and hind legs back Light-Yellow Ocher (13) with Raw Umber (280) dashes, and bordered with a wide Raw Umber (280) band; white stipples on the lateral surface of the head and back of the legs. The specimen IAvH-Am-17425 has the dorsal surfaces of the head, body, legs, and tail Dark Salmon (59), strongly speckled with Raw Umber (280), with greater concentration at the nape of the neck. Ventral markings or blotches on the ventral surfaces of the body and tail vary in shape and size; often with irregular margins but are consistently white or cream-colored independent of sex and age (Figs 5–7).

##### Etymology.

Named after the native human inhabitants of the Altiplano Cundiboyacense and Sabana de Bogotá. The Muiscas regarded amphibians as sacred creatures associated with sex, fertility, and the arrival of the rainy season. The specific epithet is used as a noun in apposition.

##### Distribution.

At present, *Bolitoglossamuisca* is known only from small cloud forest remnants on the western slopes of the Cordillera Oriental of Colombia in Bojacá, Granada, San Antonio del Tequendama, Silvania, and Soacha municipalities, Cundinamarca department. All specimens collected between 2390–2700 m a.s.l. (Fig. 2).

##### Natural history.

**Figure 8.**
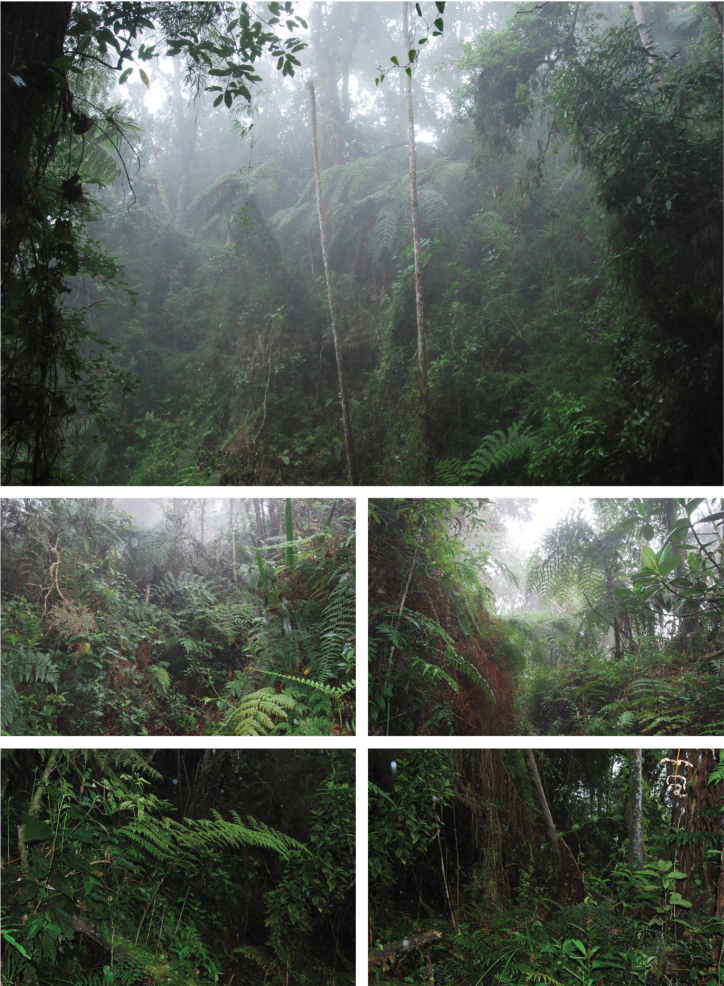
The Andean cloud forests of Bojacá, Cundinamarca, and the habitat of *Bolitoglossamuisca*. Photographs by YLP.

Individuals from Bojacá municipality were regularly found at night on the base (on the *mantillo*) and leaves of Cyatheaceae ferns, which are dominant in the cloud forests of the Tequendama region of Cundinamarca department (Fig. 8). Most individuals from San Antonio del Tequendama municipality were found active at night perching on small branches of shrubs (Araceae and Melastomataceae), usually far away from rivers or streams. During the day, a few individuals were found inactive inside bromeliads below two meters height. When handled, these salamanders produced a sticky whiteish mucoserous substance; we consider this to be a defense mechanism against potential predators (Arrivillaga and Brown 2018). Two frog species (*Pristimantis* sp. and *P.uisae*) and a lizard (*Anolisheterodermus*) were found in sympatry with *Bolitoglossamuisca*; no other salamander species were found within our fieldwork area.

##### Other material examined.

Countries are indicated in bold capitals, departments in regular capitals, municipalities, and localities in plain text. * Denotes specimens examined via photographs.

*Bolitoglossaadspersa* (*n* = 29). **Colombia**: Boyacá: Duitama, Páramo de la Rusia: ICN 4301, 4310; Toquilla, Páramo de Toquilla: ICN 9487. Cundinamarca: Bogotá, D.C., Páramo de Cruz Verde: IAvH-Am-8917, San Cristobal, Tánques de Vitelma, finca La Marranera: IAvH-Am-696, 2947, 2954, 2957-58, 2964, 2967, 2972, Usme, represa El Hato: ICN 37555; Cabrera: vereda de Hoyerías: IAvH-Am-13253 Fómeque, Laguna de Chingaza: ICN 4884, 4891; Guasca, Páramo de Guasca: ICN 4521, 4525, 4546; Fómeque, laguna Chingaza: ICN 4885; Fusagasugá, km 50 carretera Fusagasugá-La Florida: ICN 39096; Guatavita, vereda Montenquiva: IAvH-Am-13174–75, ICN 55420; Guayabetal: Páramo Atravezado: IAvH-Am-14808; Páramo de Palacio, P.N.N. Chingaza: IAvH-Am-3101, 3096, Quétame, km 22 carretera central Villavicencio-Alto del Tigre: ICN 7123; Ubaque, reserva ecológica Matarredonda: IAvH-Am-13254.

*Bolitoglossacapitana* (*n* = 4). **Colombia**: Cundinamarca: Albán, Granjas del Padre Luna: ICN 9221 & MLS 182–184*.

*Bolitoglossaguaneae* (*n* = 27). **Colombia**: Santander: Charalá, Virolín, Cañaverales, sector El Reloj: ICN 5197, 8555, 8557, 12770–72, 34230, Cuchilla del Fara: ICN 47980, Hacienda La Sierra: ICN 34229–30, km 56: ICN 19558, UIS-A 1369, UIS-A 2078, UIS-A 2082, UIS-A 2179, UIS-A 2317, UIS-A 2325–6, UIS-A 2891, UIS-A 2893, UIS-A 2895, UIS-A 2898–9, paratypes UIS-A 2203, UIS-A 2320, UIS-A 2324, UIS-A 2897.

*Bolitoglossamuisca* (*n* = 54). **Colombia**: Cundinamarca: Bojacá, vereda Roble Hueco, predio La Esmeralda: IAvH-Am-17413–22, Predio Peñas Blancas: IAvH-Am-17423–28; Granada, hacienda El Soche: ICN 3544–48, MVZ 167997*; San Antonio del Tequendama, vereda Roquemonte, cerca de entrada Parque Chicaque: ICN 60319–25; Silvania, vereda Noruega Alta, Hacienda La Tribuna: ICN 58245–58268; Soacha, vereda Cascajal: IAvH-Am-17429.

*Bolitoglossanicefori* (*n* = 13). **Colombia**: Santander: Floridablanca, El Mortiño, quebrada Torrentosa: ICN 50000, 58227, 58231; Zapatoca, finca Los Puentes, quebrada Uchuvala: ICN 58223–24, 58226; Piedecuesta, vereda Los Monos: UIS-A 2987, UIS-A 2991; Los Santos, vereda El Carrizal, finca Utopía: UIS-A 5273–4; Guapotá, vereda Las Flores, finca La Chocolatera: UIS-A 5264; San Gil, vereda San José, finca La Esperanza: UIS-A 5270–1.

*Bolitoglossapandi* (*n* = 20). **Colombia**: Cundinamarca: Pandi, vereda Buenos Aires Alta: ICN 45500, Supatá, vereda Las Lajas, reserva Cuzcungos: ICN 58492–05, Villeta, La Esmeralda: IAvH-Am-10303–08.

## ﻿Discussion

### ﻿Taxonomic background

Brame and Wake (1963: 44) examined a single specimen of *Bolitoglossa* from the Tequendama region of Cundinamarca (ICNB Tequendama), which was regarded as an undescribed species morphologically similar to *B.adspersa*. Despite finding diagnostic morphological differences between this specimen and those from *B.adspersa*, the limited sample size precluded any attempt by these authors to describe it as a new species. Our morphological and molecular results support Brame and Wake’s (1963) hypothesis. Through our conversations with Giovanni Chaves-Portilla we came across an unpublished manuscript written by the late David B. Wake and Arden H. Brame, dated sometime between the late 1980s and early 1990s (John D. Lynch pers. comm.), in which they proposed descriptions of three new *Bolitoglossa* from Colombia. One of these new species corresponds to *Bolitoglossamuisca* and is known from a site called Hacienda ‘El Soche’, Granada municipality, Cundinamarca department, Colombia, 2600 m a.s.l., i.e., from the same locality of MVZ 167997 (Fig. 1). Wake and Brame had planned to designate four types for this new species housed at Colección de Anfibios, Instituto de Ciencias Naturales, Universidad Nacional de Colombia, Bogotá D.C., namely ICN 3544–46 and ICN 3550. We managed to examine these specimens and confirm them to be conspecific with *Bolitoglossamuisca*, but unfortunately these are not in the best condition and are therefore designated as referred material.

### ﻿Phylogenetic status

Our taxonomic sampling indicates that *Bolitoglossamuisca* is sister to *B.adspersa* and both are reciprocally monophyletic (Fig. 1). These two species are close geographically and their genetic uncorrected p-distance for the 16S and cyt b (7.0%) fragments were relatively low (2.1 and 7%, respectively). Yet, lower distances between morphologically well-defined sister species of *Bolitoglossa* have been previously reported (Batista et al. 2014; Meza-Joya et al. 2017), with the smallest divergence reaching 0.5% for 16S (Parra-Olea et al. 2004). In addition, species delimitation analyses provided support to the distinctiveness of *B.muisca* as a new species.

### ﻿Threats and conservation status

Deforestation, logging, and forest clearing are the main threats faced by the habitat of the new species in the remnant cloud forests of the Tequendama region in Cundinamarca department, Colombia. Nonetheless, the type locality of *Bolitoglossamuisca* is located within the regional protected area ‘Distrito de Manejo Integrado Cerro Manjui – Salto del Tequendama’, a conservation project led by Empresas Públicas de Medellín (EPM) and Fundación Natura that focuses on improving connectivity among cloud forests remnants of the Tequendama region. The calculated Extent of Occurrence (EOO) using the localities where the species is distributed is 102 km^2^ (estimate made with GeoCAT; Bachman et al. 2011). *Bolitoglossamuisca* is only known from an area of 102 km^2^ and only the type locality has a certain degree of protection. However, based on our results and field observation we consider that this species should be considered as Endangered (EN) using the IUCN criteria B1b(iii) of the IUCN, given its small known range (< 5000 km), and the current threats to its native habitat. The consequent loss of native vegetation may be causing the new species described here to be most likely threatened by habitat loss, and a monitoring program is warranted to better assess the current status of its few known populations (Liu et al. 2022).

## Supplementary Material

XML Treatment for Bolitoglossa muisca

## ﻿Appendix 1

**Table A1.** GenBank accession numbers of the *Bolitoglossa* and outgroup (*) sequences used in the phylogenetic analysis. Accession numbers in bold correspond to the new sequences presented in this paper. Subspecies and species group sensu Parra-Olea et al. (2004).

Species	Subspecies	Group	16S	cyt *b*	Rag–1	Voucher	Locality
*B.adspersa*	*Eladinea*	*adspersa*	OQ681031	OQ685920	–	MLC348	Colombia: Cundinamarca, La Calera
*B.adspersa*	*Eladinea*	*adspersa*	AF218492	AF212984	–	MVZ 158485	Colombia: Cundinamarca, Ubaté
*B.adspersa*	*Eladinea*	*adspersa*	OQ681032	–	–	N/A	Colombia: Cundinamarca, Choachí
*B.adspersa*	*Eladinea*	*adspersa*	OQ681033	–	–	DEQ4	Colombia: Santander, Sucre
*B.altamazonica*	*Eladinea*	*adspersa*	MT301583	MT301731	MT301938	ORP 505	Peru: Loreto, near Nauta
*B.altamazonica*	*Eladinea*	*adspersa*	MT301579	MT301799	MT301913	F 01	Peru: Loreto, near Nauta
*B.awajun*	*Eladinea*	*adspersa*	MG944411	MG944420	MG944441	CRBIIAPAR1124	Peru: San Martín, San Martín, San Antonio, Cordillera Escalera
*B.awajun*	*Eladinea*	*adspersa*	MG944412	MG944422	MG944442	CRBIIAPAR1125	Peru: San Martín, San Martín, San Antonio, Cordillera Escalera
*B.biseriata*	*Eladinea*	*adspersa*	AY526118	AY526161	–	S13236	Panamá: Kuna Yala, Nusagandi
*B.biseriata*	*Eladinea*	*adspersa*	–	AY526161	KC614436	MVZ 232943	Panamá: Kuna Yala, Nusagandi
*B.caldwellae*	*Eladinea*	*adspersa*	AY526129	AY526168	–	MPEG 12881	Brazil: Acre, Porto Walter
*B.caldwellae*	*Eladinea*	*adspersa*	MT301584	–	–	CFBHT 54	Brazil: Acre, Serra do Divisor
*B.chucantiensis*	*Eladinea*	*adspersa*	KM527324	–	–	SMF 97141	Panamá: Darién, Chepigana, Cerro Chucantí
*B.equatoriana*	*Eladinea*	*adspersa*	MT301585	MT301789	–	IIAP999	Peru: Loreto, Maynas, Curaray river
*B.equatoriana*	*Eladinea*	*adspersa*	–	DQ353842	KC614451	QCAZ 25448	Ecuador: Napo, Estación Biológica Jatun Sacha
*B.guaneae*	*Eladinea*	*adspersa*	KU985264	KX458162	–	UIS-A 5275	Colombia: Santander, Charalá, Virolín
*B.guaneae*	*Eladinea*	*adspersa*	KU985265	KX458163	–	UIS-A 5276	Colombia: Santander, Charalá, Virolín
*B.hypacra*	*Eladinea*	*adspersa*	MT301588	–	–	SAS 446	Colombia: Antioquia, Paramo Frontino
*B.hypacra*	*Eladinea*	*adspersa*	MT301589	–	–	SAS 447	Colombia: Antioquia, Paramo Frontino
*B.leandrae*	*Eladinea*	*adspersa*	KC257102	–	–	MCNUP 63	Colombia: Norte de Santander, San Antonio
*B.leandrae*	*Eladinea*	*adspersa*	MT301592	MT301732	MT301921	VVO 837	Colombia: Meta, Villavicencio
*B.lozanoi*	*Eladinea*	*adspersa*	KU985266	KX458164	–	H 3	Colombia: Santander, Floridablanca
*B.lozanoi*	*Eladinea*	*adspersa*	KU985267	KX458165	–	UIS-A 5269	Colombia: Santander, Girón
*B.madeira*	*Eladinea*	*adspersa*	AY526128	AY526167	–	LSUMZ-H 3086	Brazil: Amazonas, Ituxi river, Madeireira Scheffer
*B.madeira*	*Eladinea*	*adspersa*	MT301594	MT301733	MT301895	MPEG 28601	Brazil: Acre: Fazenda Experimental Catuaba, Branco river
*B.medemi*	*Eladinea*	*adspersa*	AY526123	AY526163	KC614437	S13237	Panamá: Kuna Yala, Nusagandi
*B.medemi*	*Eladinea*	*adspersa*	KM527327	–	–	SMF 97131	Panamá: Darién, Serranía de San Blas
*B.mucuyensis*	*Eladinea*	*adspersa*	JN635335	JQ665282	–	CVULA 7100	Venezuela: Mérida, La Mucuy
*B.mucuyensis*	*Eladinea*	*adspersa*	JN635336	–	–	CVULA 7101	Venezuela: Mérida, La Mucuy
*B.nicefori*	*Eladinea*	*adspersa*	KX458176	KX458166	–	UIS-A 5270	Colombia: Santander, San Gil
*B.nicefori*	*Eladinea*	*adspersa*	KX458177	KX458167	–	UIS-A 5271	Colombia: Santander, San Gil
*B.orestes*	*Eladinea*	*adspersa*	JN635340	JQ665280	–	CVULA 7109	Venezuela: Mérida, Sierra La Culata
*B.orestes*	*Eladinea*	*adspersa*	JN635351	JQ665281	–	MC00	Venezuela: Mérida, Sierra La Culata
*B.palmata*	*Eladinea*	*adspersa*	AY526125	AY526164	–	KU 217422	Ecuador: Napo, Cordillera de Guacamayos
*B.palmata*	*Eladinea*	*adspersa*	MT301595	–	MT301941	MZUTI 2220	Ecuador: Napo, Cordillera de los Guacamayos, La Virgen
*B.paraensis*	*Eladinea*	*adspersa*	MT301596	–	–	CFBHT 20324	Brazil: Para, Moju, Moju river
*B.paraensis*	*Eladinea*	*adspersa*	MT301597	MT301734	MT301892	MPEG 31672	Brazil: Para, Santa Izabel, Semente Etérea, Vila do Carapuru
*B.peruviana*	*Eladinea*	*adspersa*	MG944408	MG944417	MG944436	CRBIIAP AR1118	Peru: Loreto, Alto Amazonas, Cordillera Escalera
*B.peruviana*	*Eladinea*	*adspersa*	MT301600	MT301791	MT301900	CRBIIAP AR1038	Peru: Loreto, Alto Amazonas, Balsa Puerto, Shawi
*B.muisca*	*Eladinea*	*adspersa*	AY526135	AY526173	–	MVZ 167997	Colombia: Cundinamarca, El Soche
*B.muisca*	*Eladinea*	*adspersa*	OQ681034	–	–	IAvH-Am-17414	Colombia: Cundinamarca, Bojacá
*B.muisca*	*Eladinea*	*adspersa*	OQ681035	–	–	IAvH-Am-17417	Colombia: Cundinamarca, Bojacá
*B.sima*	*Eladinea*	*adspersa*	AY526134	AY526172	–	MVZ 2057	Colombia: Valle del Cauca
*B.* sp. ‘Chilma’	*Eladinea*	*adspersa*	–	KC614431	KC614456	QCAZ 39981	Ecuador: Carchi, Chilma Bajo
*B.* sp. ‘Cisneros’	*Eladinea*	*adspersa*	MT301603	MT301735	MT301876	AFJ 006	Colombia: Valle del Cauca, Buenaventura, Cisneros, Los Turbos
*B.* sp. ‘Cisneros’	*Eladinea*	*adspersa*	MT301602	–	–	AFJ 010	Colombia: Valle del Cauca, Buenaventura, Cisneros, Los Turbos
*B.* sp. ‘Jingurudó’	*Eladinea*	*adspersa*	KM527329	–	–	MHCH 2663	Panamá: Darién, Chepigana, Serranía de Jingurudó
*B.* sp. ‘Llullapichi’	*Eladinea*	*adspersa*	MT301618	MT301741	MT301898	FGZC 4837	Peru, Huánuco, Panguana station, lower Llullapichis river
*B.* sp. ‘Llullapichi’	*Eladinea*	*adspersa*	MT301614	MT301739	MT301905	CORBIDI 14387	Peru: Huanuco, Puerto Inca, Llullapichis: Cordillera del Sira
*B.* sp. ‘Pensilvania’	*Eladinea*	*adspersa*	MT301607	MT301793	MT301917	GGD 640	Colombia: Caldas, Pensilvania, road to Arboleda
*B.* sp. ‘Pirinari’	*Eladinea*	*adspersa*	MT301700	–	–	MUBI 10081	Peru: Loreto, Parinari, Hamburgo, Samiria river
*B.* sp. ‘Pirinari’	*Eladinea*	*adspersa*	MT301701	MT301778	MT301885	MUBI 10099	Peru: Loreto, Parinari, Pithecia, Samiria river
*B.* sp. ‘Salamina’	*Eladinea*	*adspersa*	MT301606	MT301736	MT301875	GGD 111	Colombia: Caldas, Salamina, Tribunas farm
*B.* sp. ‘Sanare’	*Eladinea*	*adspersa*	MT301608	MT301737	MT301926	MOE 01	Venezuela: Lara, Sanare, El Blanquito, Yacambu National Park
*B.* sp.	*Eladinea*	*adspersa*	MT301604	MT301792	–	DQ 175	Venezuela
*B.* sp.	*Eladinea*	*adspersa*	MT301605	–	MT301871	DQ 177	Venezuela
*B.tamaense*	*Eladinea*	*adspersa*	KC257098	–	–	MCNUP 56	Colombia: Norte de Santander, Los Remansos
*B.tamaense*	*Eladinea*	*adspersa*	KC257099	–	–	MCNUP 57	Colombia: Norte de Santander, Los Remansos
*B.tapajonica*	*Eladinea*	*adspersa*	MT301711	MT301802	MT301933	MPEG 31688	Brazil: Para, Juruti
*B.tapajonica*	*Eladinea*	*adspersa*	MT301712	MT301786	MT301934	MPEG 31695	Brazil: Para, Lorena
*B.taylori*	*Eladinea*	*adspersa*	KM527337	–	–	AB 171	Panamá: Darién, Pinogana, Serranía de Pirre
*B.taylori*	*Eladinea*	*adspersa*	KM527335	–	–	AB 173	Panamá: Darién, Pinogana, Serranía de Pirre
*B.vallecula*	*Eladinea*	*adspersa*	MT301713	MT301787	MT301874	AFJ 48	Colombia: Valle del Cauca, El Cairo, Cerro del Inglés
*B.walkeri*	*Eladinea*	*adspersa*	MT301714	MT301788	MT301873	AFJ 02	Colombia: Valle del Cauca, Cali, San Antonio
*B.walkeri*	*Eladinea*	*adspersa*	MT301715	–	–	AFJ 03	Colombia: Valle del Cauca, Cali, San Antonio
*B.yariguiensis*	*Eladinea*	*adspersa*	KU985275	KX458173	–	UIS-A 5280	Colombia: Santander, San Vicente de Chucurí
*B.yariguiensis*	*Eladinea*	*adspersa*	KU985276	KX458174	–	UIS-A 5281	Colombia: Santander, San Vicente de Chucurí
*B.cerroensis**	*Eladinea*	*epimela*	AF199233	AF199195	KC614459		
*B.epimela**	*Eladinea*	*epimela*	AY526120	AF212097	–		
*B.minutula**	*Eladinea*	*epimela*	AY526124	AF212098	KC614434		
*B.nigrescens**	*Eladinea*	*schizodactyla*	JQ899164	JQ899194	–		
*B.robusta**	*Eladinea*	*schizodactyla*	EU448109	EU448110	–		
*B.schizodactyla**	*Eladinea*	*schizodactyla*	AY526133	AY526171	–		
*B.splendida**	*Eladinea*	*subpalmata*	JQ899150	JQ899181	–		
*B.subpalmata**	*Eladinea*	*subpalmata*	AF416697	AF212094	–		
*B.tica**	*Eladinea*	*subpalmata*	JQ899162	JQ899192	–		
